# Formation of Biogenic Manganese Oxide Nodules on Hyphae of a New Fungal Isolate of *Periconia* That Immobilizes Aqueous Copper

**DOI:** 10.1264/jsme2.ME23102

**Published:** 2024-06-12

**Authors:** Shihori Tsushima, Yuma Nishi, Ryo Suzuki, Masaru Tachibana, Robert A. Kanaly, Jiro F. Mori

**Affiliations:** 1 Graduate School of Nanobioscience, Yokohama City University, Japan

**Keywords:** manganese-oxidizing fungus, *Periconia*, biogenic Mn oxides, heavy metals

## Abstract

Mn(II)-oxidizing microorganisms are considered to play significant roles in the natural geochemical cycles of Mn and other heavy metals because the insoluble biogenic Mn oxides (BMOs) that are produced by these microorganisms adsorb other dissolved heavy metals and immobilize them as precipitates. In the present study, a new Mn(II)-oxidizing fungal strain belonging to the ascomycete genus *Periconia*, a well-studied plant-associating fungal genus with Mn(II)-oxidizing activity that has not yet been exami­ned in detail, was isolated from natural groundwater outflow sediment. This isolate, named strain TS-2, was confirmed to oxidize dissolved Mn(II) and produce insoluble BMOs that formed characteristic, separately-located nodules on their hyphae while leaving major areas of the hyphae free from encrustation. These BMO nodules also adsorbed and immobilized dissolved Cu(II), a model analyte of heavy metals, as evidenced by elemental mapping ana­lyses of fungal hyphae-BMO assemblages using a scanning electron microscope with energy-dispersive X-ray spectroscopy (SEM-EDX). Analyses of functional genes within the whole genome of strain TS-2 further revealed the presence of multiple genes predicted to encode laccases/multicopper oxidases that were potentially responsible for Mn(II) oxidation by this strain. The formation of BMO nodules may have functioned to prevent the complete encrustation of fungal hyphae, thereby enabling the control of heavy metal concentrations in their local microenvironments while maintaining hyphal functionality. The present results will expand our knowledge of the physiological and morphological traits of Mn(II)-oxidizing *Periconia*, which may affect the natural cycle of heavy metals through their immobilization.

Some microorganisms have the ability to solubilize or immobilize heavy metals and are, thus, considered to play significant roles in the natural geochemical cycles of these metals (White *et al.*, 1997; Gadd and Raven, 2010). Manganese (Mn) is the second most abundant transition metal in the Earth’s crust after iron (Fe) and is commonly found as Mn(IV) oxide minerals in terrestrial and aquatic environments (Post, 1999). The formation of insoluble Mn oxides is induced through the oxidation of soluble Mn(II) to Mn(III) and Mn(IV), which is a thermodynamically favorable process that is extremely slow at neutral pH (Morgan, 2005). A number of microorganisms, including bacteria and fungi, have the ability to oxidize dissolved Mn(II) and immobilize it as Mn oxides and, thus, they are regarded as the primary players in the natural redox cycling of Mn because biological Mn(II) oxidation rates are up to 10^5^-fold faster than abiotic processes (Hastings and Emerson, 1986; Spiro *et al.*, 2010; Tebo *et al.*, 2010).

In fungi, the ability to oxidize Mn(II) to Mn(III/IV) has been detected in the phyla *Ascomycota* and *Basidiomycota* (Miyata *et al.*, 2007; Zhou and Fu, 2020). Mn(II)-oxidizing ascomycetes that belong to the orders *Pleosporales* (*e.g.*, the‍ ‍genera *Alternaria*, *Leptosphaerulina*, *Paraconiothyrium*, *Periconia*, *Phoma*, *Pleosporales*, *Pithomyces*, *Pyrenochaeta*, and *Stagonospora*), *Hypocreales* (*e.g.*, the genus *Acremonium*), and *Capnodiales* (*e.g.*, the genus *Cladosporium*) were found in and isolated from natural freshwater, sediments, soils, and mining sites (Takano *et al.*, 2006; Santelli *et al.*, 2010; Tang *et al.*, 2013; Carmichael *et al.*, 2015; Wang *et al.*, 2022; Xu *et al.*, 2023). The physiological reason(s) for fungal Mn(II) oxidation remains unclear; previous studies hypothesized that microbial Mn(II) oxidation may be induced unintentionally during extracellular superoxide production or may be related to lignocellulose degradation and/or protection from external stresses even though these microorganisms do not gain energy from Mn(II) oxidation reactions (Tebo *et al.*, 2004; Santelli *et al.*, 2010; Hatakka and Hammel, 2011; Hansel *et al.*, 2012).

The Mn oxide minerals produced by microorganisms, so-called biogenic Mn oxides or BMOs, adsorb other heavy metal ions and precipitate them due to their poorly crystalline or nano-sized structures (Post, 1999; Kim *et al.*, 2003; Tani *et al.*, 2004; Sasaki *et al.*, 2008; Duckworth *et al.*, 2017). Therefore, Mn(II)-oxidizing microorganisms in nature are considered to play significant roles in the immobilization of not only Mn, but also heavy metals, such as Co, Ni, Cu, Zn, Cd, and Pb, in aquatic environments (Nelson *et al.*, 1999; Tebo *et al.*, 2004; Xu *et al.*, 2023), and also have potential as biotechnologically useful tools for the biosorption and bioremediation of toxic heavy metal pollutants (Brierley, 1990; Piazza *et al.*, 2019). The application of fungi may provide some advantages over bacteria, such as their resistance to changes in growth conditions and higher operational efficiencies to recover immobilized mineral precipitates on their mycelia (Bayramoğlu *et al.*, 2003; Sasaki *et al.*, 2006; Wang *et al.*, 2022). Fungal BMOs were previously shown to be deposited on the cellular structures of fungi, which resulted in the coating of BMOs along their hyphae, or on extracellular polymers produced by the fungi (Santelli *et al.*, 2011; Tang *et al.*, 2013). It is also important to note that groups of fungi exhibited tolerance to excess heavy metal exposure through the chelation of metals by excreted ligands or cell-wall material (Bellion *et al.*, 2006; Anahid *et al.*, 2011). These findings have motivated researchers to discover novel Mn(II)-oxidizing fungal strains from different environments and characterize their physicochemical, physiological, and morphological properties.

A new strain of Mn(II)-oxidizing ascomycete fungus belonging to the order *Pleosporales*, *Periconia* sp. strain TS-2, was isolated from natural freshwater outflow sediment and the whole genome sequence of this strain was obtained (Tsushima *et al.*, 2023). This strain is the second known member within the genus *Periconia* with the ability to oxidize Mn(II), following strain SM10a2_F1 (Xu *et al.*, 2023), and represents the first genomic information on the Mn(II)-oxidizing members of this genus. The aims of the present study were to investigate the ability of strain TS-2 to oxidize dissolved Mn(II), characterize the morphological traits of fungal hyphae-BMO associations, and demonstrate the immobilization of other heavy metals on fungal hyphae in association with BMO production through scanning electron microscope with energy-dispersive X-ray spectoscopy (SEM-EDX) elemental mapping analysis by using copper [Cu(II)] - Cu(II) was selected as the model analyte to study‍ ‍heavy metal adsoption on BMOs (Zhang *et al.*, 2015; Park *et al.*, 2020). The present results will expand our understanding of the physiological and morphological traits of Mn(II)-oxidizing fungi and their potential to immobilize other heavy metals coupled with their production of BMOs.

## Materials and Methods

### Microbial isolation and culture conditions of strain TS-2

The fungal isolate strain TS-2 was obtained through the screening of Mn(II)-oxidizing microorganisms from the sediment of a natural groundwater outflow in Sakae-ku, Yokohama, Japan (Tsushima *et al.*, 2023). This outflow is one of the headstreams of a local river (Itachi River) that was shown to have dissolved Mn(II) concentrations >1‍ ‍mg L^–1^ originating from a natural spring. In May 2021, surface sediment was collected from the outflow into a sterilized 50-mL centrifugation tube and was used as the microbial inoculation source. The concentration of dissolved Mn(II) in outflow water was assessed using a spectrophotometer (DR 3900; HACH) by the periodate oxidation method (HACH method 8034). To screen for microorganisms with the ability to oxidize Mn(II), outflow sediment was suspended in sterilized water and spread on modified PC agar plates, which contained 40‍ ‍mg L^–1^ MnSO_4_·5H_2_O (=179.5‍ ‍μM Mn[II]), 50‍ ‍mg L^–1^ yeast extract, and 2% agarose (Tyler and Marshall, 1967). Microbial colonies that exhibited a brownish color, which potentially indicated the production of BMOs, were selected for further sub-culturing and purification. BMO production by selected microorganisms was also confirmed by spotting 0.04% leucoberbelin blue (LBB) solution, which reacts with Mn(III/IV) oxides and results in a dark blue color (Krumbein and Altmann, 1973). After the screening process described above, strain TS-2 was isolated and subjected to further investigations.

### Genomic ana­lysis of strain TS-2

The whole genome sequence of strain TS-2 (GenBank accession number ASM3037842) was recently announced with details on sequencing and data processing methodologies (Tsushima *et al.*, 2023). Strain TS-2 was phylogenetically characterized through a homology search using the nucleic acid sequence of the fungal ITS region (ITS1-5.8S rRNA-ITS2). The protein-coding genes within the genome of strain TS-2 were predicted using BRAKER2 (Ver 2.1.6) (Brůna *et al.*, 2021) and these genes were functionally annotated using DIAMOND (Ver 2.1.8.162) (Buchfink *et al.*, 2015) in the blastx mode with default settings against the NCBI RefSeq fungi database.

### Mn(II) oxidation experiments

The Mn(II) oxidation and immobilization capabilities of strain TS-2 were evaluated by transferring strain TS-2 mycelia grown on agar plates into modified PC liquid medium without agarose and the concentration of dissolved Mn(II) was monitored in the cultures. One loop of strain TS-2 mycelia was taken from the edge of a colony, suspended in 50‍ ‍mL of modified PC liquid medium (pH 6.0), and incubated by rotary shaking at 50‍ ‍rpm at 25°C in the dark. During the incubation, dissolved Mn(II) concentrations in the cultures were measured using a spectrophotometer as described above and Mn(II) removal by strain TS-2 was monitored.

### XRD ana­lyses of biogenic Mn oxides

The structural characterization of the BMOs produced by strain TS-2 was performed using X-ray diffraction (XRD) ana­lysis. A mixture of fungal material and BMOs collected from a matured culture of strain TS-2 was dried and subjected to the XRD ana­lysis (Ultima IV; Rigaku) with CuKα radiation at 40 mA and 40 kV.

### Cu(II) adsorption experiments

To evaluate the adsorption behavior of dissolved heavy metals to the BMOs generated by strain TS-2, copper (Cu) was selected as the model heavy metal element and the removal of dissolved Cu(II) in strain TS-2 cultures was investigated. Strain TS-2 was pre-cultured in modified PC liquid medium with or without a Mn(II) supply for 3 days, after which 6.72‍ ‍mg L^–1^ of CuCl_2_ (=50‍ ‍μM Cu[II]) was supplied and followed by further incubations. During incubations, dissolved Cu(II) concentrations in the cultures were measured using a spectrophotometer with the bicinchoninate method (HACH method 8506) and Cu(II) immobilization by strain TS-2 was monitored.

### SEM and EDX ana­lyses

The cell morphology of strain TS-2 was exami­ned using SEM (JSM-6000; NeoScope, JEOL) with an acceleration voltage of 15‍ ‍kV. A piece of strain TS-2 mycelia grown in liquid medium was taken and dehydrated through a graded ethanol series on a polycarbonate membrane filter with a pore size of 0.2‍ ‍μm (Isopore, Merck Millipore), and was then subjected to Au sputtering before observations. To assess the adsorption of copper on the BMOs generated by strain TS-2, SEM observations (S-4300; Hitachi) with EDX (X-Max50; Horiba) of strain TS-2 mycelia incubated with Mn(II) and Cu(II) were conducted. In SEM-EDX observations, strain TS-2 mycelia dehydrated on a polycarbonate filter were coated with approximately 6.5‍ ‍nm of Pt using ion sputtering (E-1045; Hitachi) and a SEM-EDX ana­lysis was performed with an acceleration voltage of 15 kV.

## Results

### Isolation of Mn(II)-oxidizing fungal strain TS-2

**Fig. 1.**
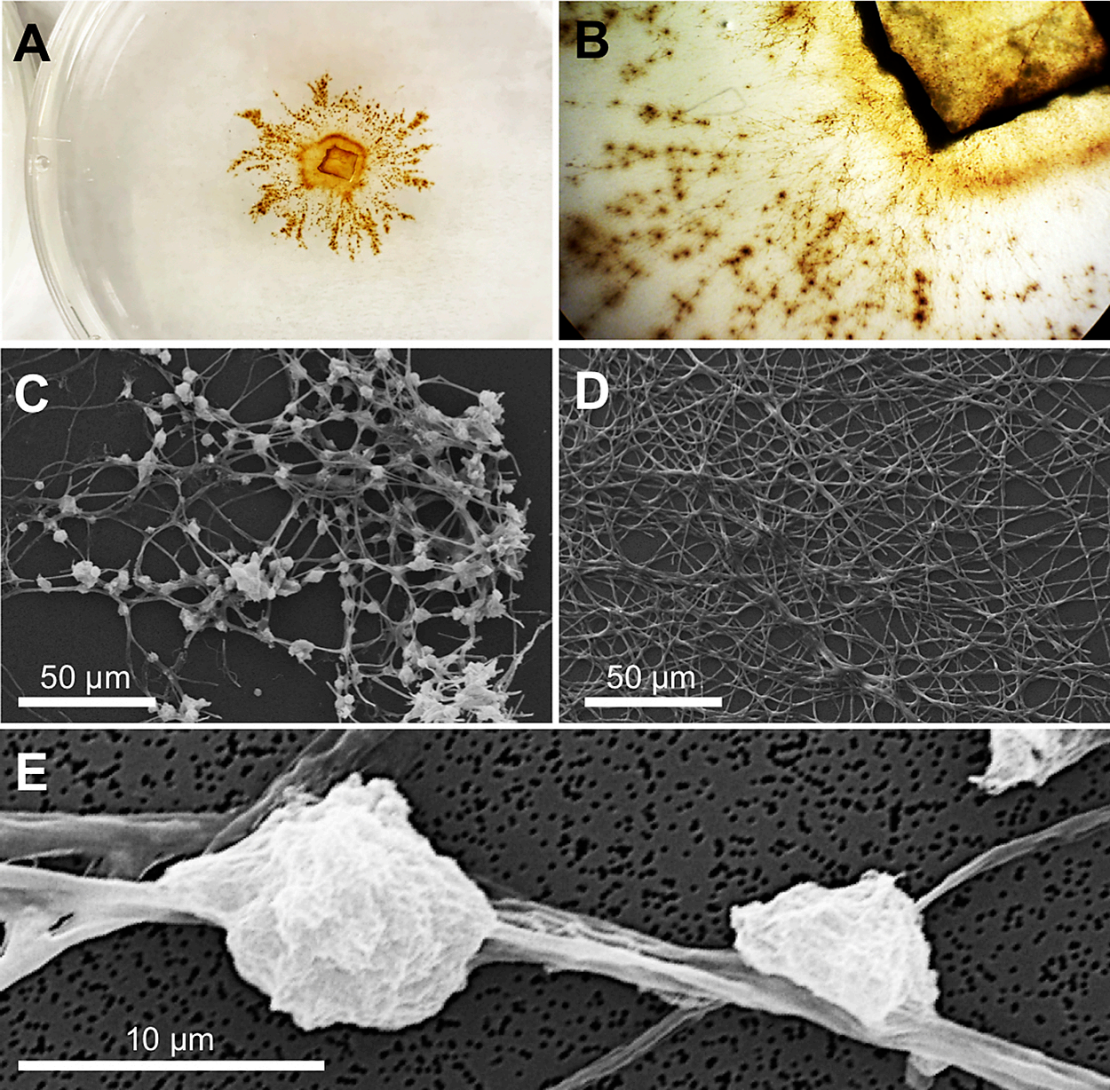
Morphology of Mn(II)-oxidizing fungal strain TS-2. (**A, B**) Photographs of a colony of strain TS-2 grown on an agar plate in a 9-cm Petri dish that produced brownish precipitates on its hyphae. (**C, D, and E**) SEM images of 10-day-old matured strain TS-2 mycelia that grew in liquid media with (**C**) or without (**D**) Mn(II); a magnified image of the nodules on hyphae (**E**).

**Fig. 2.**
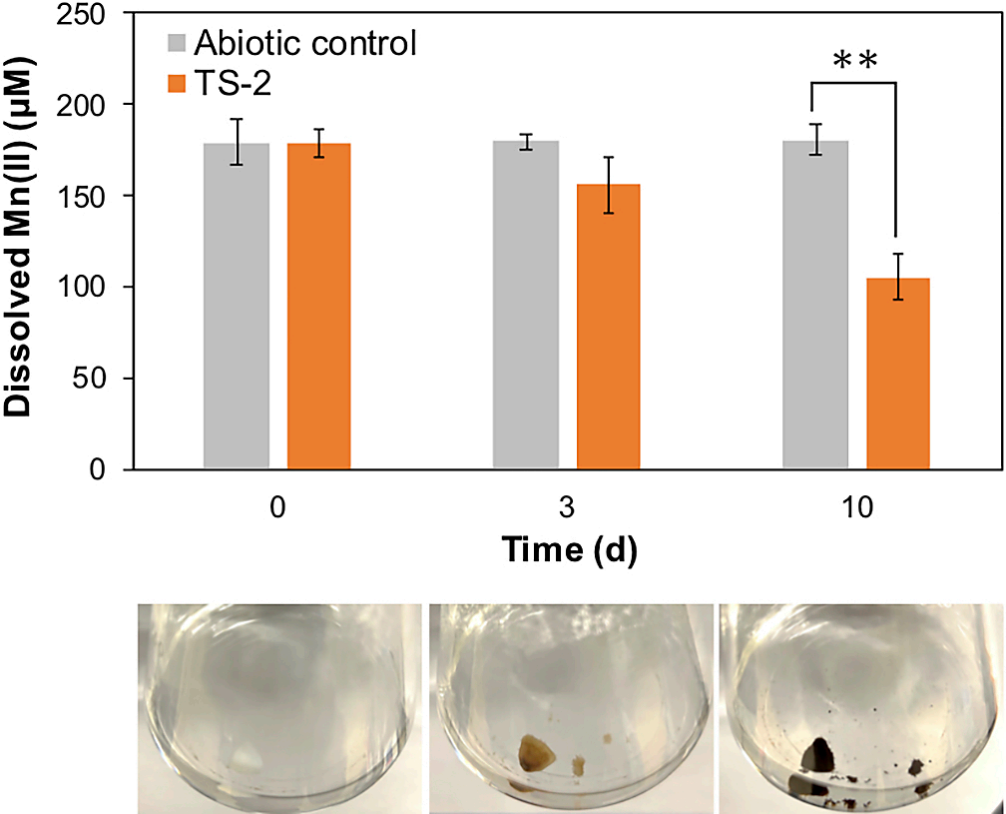
Dissolved Mn(II) concentration changes in strain TS-2 cultures incubated in modified PC liquid medium over a 10-day incubation period (days 0, 3, and 10 of the incubation). Strain TS-2 cultures (orange bars) and abiotic controls without the strain TS-2 inoculant (gray bars) were prepared in triplicate. **P<0.01 (Welch’s *t*-test), error bars indicate standard deviations (*n*=3). Photographs of strain TS-2 mycelia incubated in 50‍ ‍mL of liquid medium in a 200-mL volume conical flask taken at each time point are shown at the bottom.

Strain TS-2 was isolated from groundwater outflow, which contained 1.5‍ ‍mg L^–1^ dissolved Mn(II) when sampling was conducted. Colonies of strain TS-2 appeared to grow on the modified PC plate exhibiting the accumulation of brownish precipitates on their hyphae (Fig. 1A and B), and were purified by sub-culturing. The ITS region of strain TS-2 was the most closely related to *Periconia macrospinosa* MR30-1 (KT220671; 100% identity) and *P. macrospinosa* SCAU-F-210 (KF881777; 99.3%), indicating that strain TS-2 is affiliated with the genus *Periconia* (*Ascomycota*, *Dothideomycetes*, and *Pleosporales*). When strain TS-2 was grown on the plate without a Mn(II) supply, it grew whitish hyphae without brownish precipitates. SEM observations showed that the brownish precipitates produced by strain TS-2 formed nodules <10‍ ‍μm on hyphae (Fig. 1C), but not in the absence of Mn(II) (Fig. 1D). Each mineral nodule appeared to be separately located, leaving larger areas of hyphae uncovered (Fig. 1E). When a piece of strain TS-2 mycelia grown on the plate without Mn(II) for 3 days was transferred into modified PC liquid medium and incubated, it produced brownish precipitates on mycelia by day 3 of the incubation (Fig. 2). Corresponding to the color change in strain TS-2 mycelia, these liquid cultures showed a decrease in dissolved Mn(II) by day 3, which reached approximately 58% of the initial concentration in the matured culture after 10 days of the incubation; this decrease was not observed in the abiotic control, which did not contain strain TS-2 mycelia (Fig. 2).

### Chemical characterization of brownish precipitates produced by strain TS-2

**Fig. 3.**
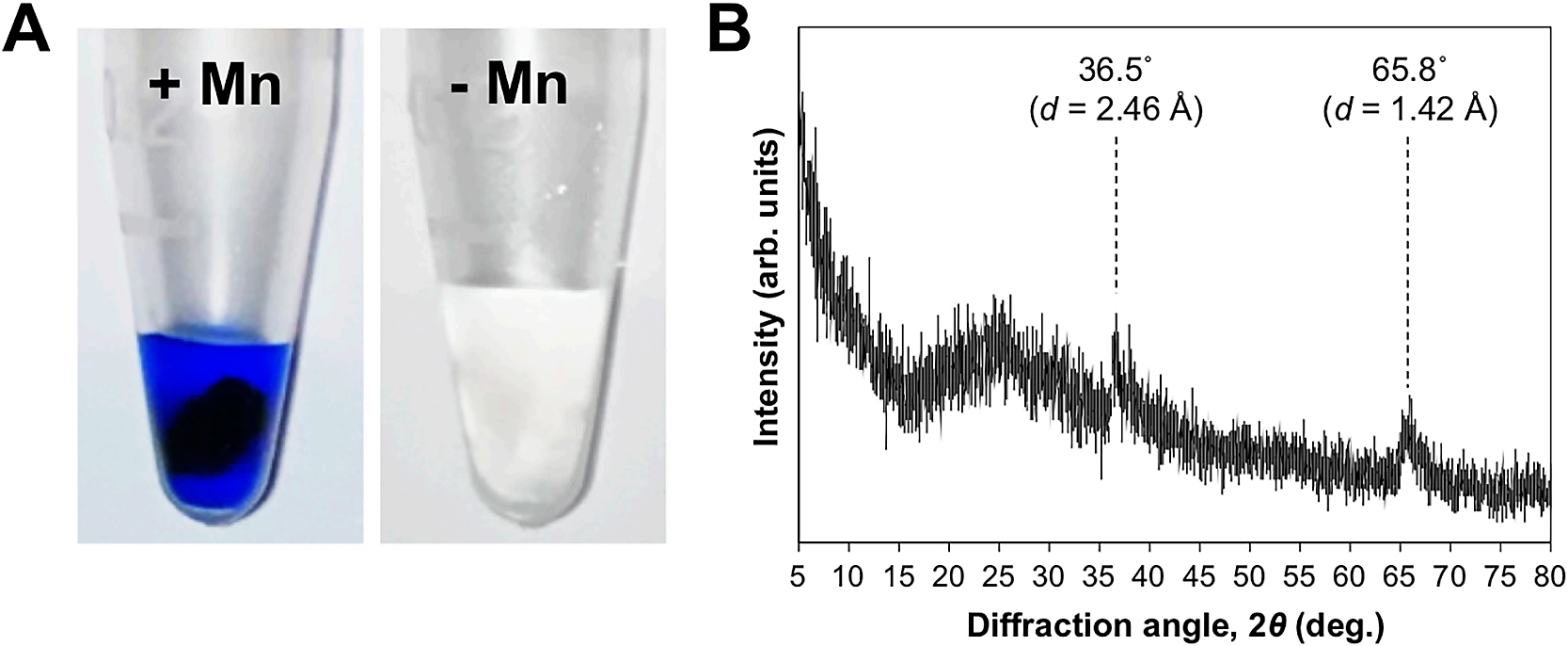
LBB assay results of strain TS-2 mycelia incubated with (left) or without (right) a Mn(II) supply (**A**) and the XRD spectrum of BMOs produced by strain TS-2 (**B**). In the XRD spectrum, the positions of two broad peaks at 36.5° (*d*=2.46 Å) and 65.8° (*d*=1.42 Å) were indicated, which represented the known typical patterns of poorly crystalline BMOs.

To establish whether the brownish precipitates produced by strain TS-2 consisted of Mn(III/IV) oxides, a piece of strain TS-2 mycelia with or without brownish precipitates taken from a liquid culture incubated for 10 days was subjected to the LBB assay. The results obtained showed that mycelia with precipitates were dark blue in color, whereas a color change was not observed for mycelia incubated without Mn(II) (Fig. 3A). The further structural characterization of precipitates produced in the strain TS-2 culture by the XRD ana­lysis revealed two weak broad peaks at 36.5° (*d*=2.46 Å) and 65.8° (*d*=1.42 Å) (Fig. 3B), which corresponded to the typical XRD patterns of BMOs that consisted of poorly crystalline vernadite (δ-MnO_2_), a variety of birnessite (Post, 1999; Tani *et al.*, 2004; Miyata *et al.*, 2006; Wang *et al.*, 2022).

### Identification of functional genes of strain TS-2 that are potentially related to Mn(II) oxidation

Previous studies indicated that fungal laccases/multicopper oxidases play a crucial role in Mn(II) oxidation in fungi (Höfer and Schlosser, 1999; Tebo *et al.*, 2010; Janusz *et al.*, 2013; Tojo *et al.*, 2020; Zeiner *et al.*, 2021). Therefore, the functional genes predicted to encode these enzymes were screened across the genome of strain TS-2 and 14 genes were identified as putative laccase/multicopper oxidase (548–662 aa)-encoding genes based on their homologies with known enzymes from various fungal groups (Table S1).

### Copper immobilization capability in strain TS-2

**Fig. 4.**
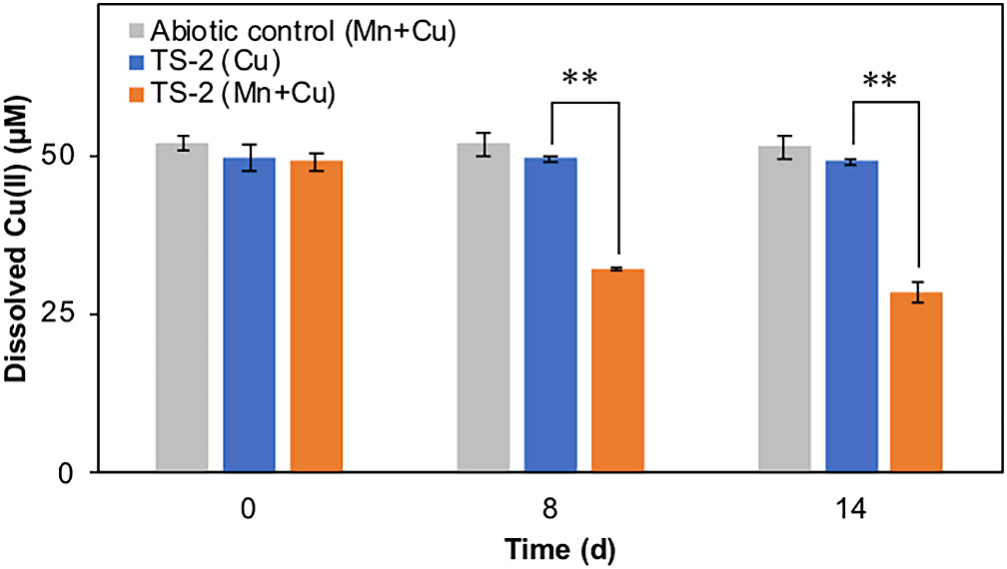
Dissolved Cu(II) concentration changes in strain TS-2 cultures pre-incubated with (orange) or without (blue) Mn(II) and incubated with Cu(II) for a 2-week incubation period. Strain TS-2 cultures in each treatment and the abiotic control that contained Mn(II) and Cu(II), but not strain TS-2 (gray) were prepared in triplicate. **P<0.01 (Welch’s *t*-test), error bars indicate standard deviations (*n*=3).

A colony of strain TS-2 that was pre-incubated in modified PC liquid medium for 3 days and produced BMOs was further exposed to 50‍ ‍μM Cu(II) and incubated, resulting in a significant decrease in dissolved Cu(II) that reached approximately 57% of the initial concentration after 14 days of the incubation (Fig. 4). This significant decrease in dissolved Cu(II) was not observed in the abiotic control that contained Mn(II) and Cu(II) or in the strain TS-2 culture that was pre-incubated without Mn(II), in which the initial concentration of Cu(II) decreased by less than 1% after the incubation (Fig. 4).

**Fig. 5.**
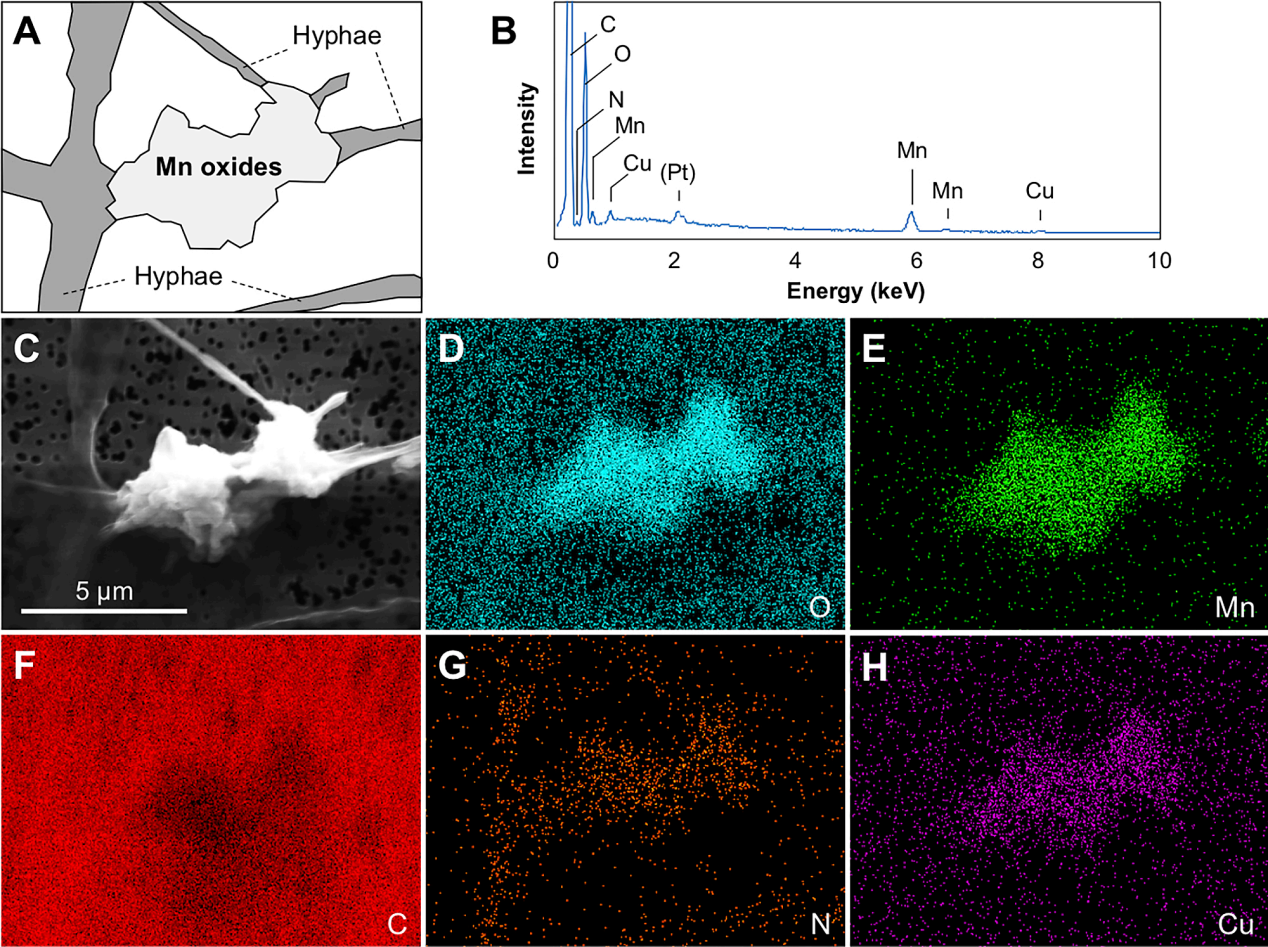
An SEM image and EDX elemental mapping of a representative Mn oxide precipitate on hyphae of strain TS-2. (**A**) Illustration of the EDX observed field; (**B**) EDX spectrum of the observed field; (**C**) SEM image of the EDX observed field; Elemental mapping of (**D**) oxygen (blue), (**E**) manganese (green), (**F**) carbon (red), (**G**) nitrogen (orange), and (**H**) copper (purple).

To examine the adsorption and immobilization of copper onto BMOs, a piece of matured mycelia of strain TS-2 incubated with Mn(II) and Cu(II) for 14 days was subjected to SEM-EDX ana­lyses. In the observed field shown in Fig. 5, C, O, N, Mn, and Cu were detected as abundant elements (Fig. 5 and Table 1). In further detailed distribution ana­lyses of these abundant elements, EDX elemental mapping for the observed field that included fungal hyphae and mineral precipitates was conducted. The results obtained indicated that mineral precipitates on the hyphae of strain TS-2 were enriched with O and Mn, but contained less C than the uncovered hyphae and background polycarbonate membrane filter, which suggested that these precipitates consisted of Mn oxides (Fig. 5). Furthermore, Cu signals were enriched in accordance with the Mn oxide precipitates rather than with fungal hyphae or background signals and, thus, appeared to be adsorbed on these Mn oxides (Fig. 5).

## Discussion

### Formation of BMO nodules on hyphae of strain TS-2 via Mn(II) oxidation

The Mn(II)-oxidizing fungal isolate, strain TS-2, was obtained through screening methods using a modified agar medium based on PC medium, by which various Mn(II)-oxidizing fungal and bacterial strains were isolated from environments in previous studies (Tyler and Marshall, 1967; Ridge *et al.*, 2007; Piazza *et al.*, 2019). The results of cultivation studies on strain TS-2 followed by the monitoring of dissolved Mn(II) removal (Fig. 2), the chemical characterization of mineral precipitates produced by strain TS-2 (Fig. 3), and SEM observations of these precipitates coupled with EDX elemental mapping (Fig. 5) showed that strain TS-2 was capable of oxidizing dissolved Mn(II) and forming insoluble BMOs, even though it did not require Mn(II) for hyphal development (Fig. 1D). The BMOs produced by strain TS-2 appeared to precipitate directly on its hyphae and were not found in distant locations from hyphae, which was previously reported for Mn(II)-oxidizing *P. macrospinosa* strain SM10a2_F1 (Xu *et al.*, 2023). However, the present study revealed that the precipitates that formed were visualized by SEM as isolated nodules that were separated by larger areas of hyphae free of mineral encrustation (Fig. 1C and E). These results suggest that Mn(II) oxidation in strain TS-2 was mediated by the localized production of reactive oxygen species (ROS) or by cell wall-associated proteins (Semighini and Harris, 2008; Tang *et al.*, 2013) rather than other mechanisms, such as an association with the extracellular polymeric matrix (Zeiner *et al.*, 2021). Additional examinations on ROS production and protein distributions in strain TS-2 may aid in the identification of the mechanisms that may trigger the formation of these characteristic BMO nodules. Discontinuous BMO precipitation on hyphae has been reported for other Mn(II)-oxidizing fungal strains that belong to the genera *Pyrenochaeta* and *Acremonium* (Miyata *et al.*, 2004; Santelli *et al.*, 2011). Precipitation and hyphal encrustation with BMOs in *Pyrenochaeta* sp. strain DS3sAY3a were found to occur in association with the formation of their fruiting bodies (Santelli *et al.*, 2011), while morphological differentiation was not observed in strain TS-2 under the growth conditions used in the present study. The discontinuous precipitation of BMOs on hyphae may benefit strain TS-2 by preventing its hyphae from becoming fully encrusted with insoluble BMOs and maintaining hyphal functionality, and similar strategies were reported for other filamentous microorganisms that produce insoluble biominerals (Mori *et al.*, 2016; Gadd, 2021).

### Discovery of Mn(II)-oxidizing capability in the fungal genus *Periconia*

The fungal genus *Periconia* was first discovered in 1791 and are commonly found as plant-associated filamentous fungi (Li *et al.*, 1998; Kim *et al.*, 2004; Verma *et al.*, 2011). Members of this fungal genus have been extensively exami­ned in the field of pharmacological natural product chemistry in the past few decades, and were reported to produce various bioactive secondary metabolites, such as antimicrobial and anti-inflammatory agents (Azhari and Supratman, 2021, and references therein). However, limited information is currently available on Mn(II) oxidation in *Periconia* and their potential role in heavy metal cycling in the environment, with this study being only the second on Mn(II) oxidation in this genus after a recent study on *P. macrospinosa* strain SM10a2_F1 (Xu *et al.*, 2023). It is important to note that strains of *P. macrospinosa* were found to exhibit high laccase activities that were involved in lignin degradation (Mandyam *et al.*, 2010; Berthelot *et al.*, 2016), which is consistent with the presence of multiple genes that are predicted to encode laccases/multicopper oxidases in the genome of strain TS-2 (Table S1). Therefore, the ability to oxidize Mn(II) appears to be widely conserved within the genus *Periconia*, which may be attributed to their production of laccases and ROS, as observed in other ascomycetes (Tang *et al.*, 2013; Tojo *et al.*, 2020; Zeiner *et al.*, 2021). Further enzymatic and/or transcriptomic investigations are expected to clarify the functions of these putative laccases/multicopper oxidases in strain TS-2 and their roles in Mn(II) oxidation.

### Localized heavy metal immobilization in association with BMO production by strain TS-2

Poorly crystalline BMO precipitates on the hyphae of strain TS-2 that were generated through Mn(II) oxidation appeared to have adsorbed other co-existing dissolved heavy metals—copper in the present study—and simultaneously immobilized them on their hyphae. Copper was selected as the model analyte to study heavy metal adsorption to BMOs in the present study because it is commonly found in natural environments and was previously employed as a model analyte (Zhang *et al.*, 2015; Park *et al.*, 2020), even though the groundwater outflow where strain TS-2 originated was characterized as having trace amounts of Cu (less than 5‍ ‍μg L^–1^ in February 2019, provided by the Resources and Waste Recycling Bureau of Yokohama City via personal communications). SEM-EDX elemental mapping clearly indicated that copper localized in association with BMO precipitates (Fig. 5), and, when taken together with strain TS-2 cultures pre-incubated without Mn(II) maintaining dissolved Cu(II) concentrations (Fig. 4), provided evidence for Mn(II) oxidation and BMO precipitation processes triggering the immobilization of Cu(II) by strain TS-2. Previous studies reported that some ascomycetes have the ability to adsorb dissolved Cu(II) and accumulate it as insoluble copper sulfide particles (Caesar-Tonthat *et al.*, 1995; Hosseini *et al.*, 2012); however, copper sulfide precipitates were not identified in‍ ‍strain TS-2 cultures based on SEM-EDX elemental mapping, in which elemental sulfur was not detected as an abundant element within fungal hyphae-BMO assemblages (Fig. 5 and Table 1). Although strain TS-2 mycelia pre-incubated with Mn(II) for 3 days adsorbed dissolved Cu(II), which was subsequently supplied, when copper was supplied from the beginning of the incubation, strain TS-2 did not exhibit Mn(II) oxidation (data not shown). Similar findings showed that the presence of Cu(II) and other metal cations inhibited Mn(II) oxidation in ascomycetes by scavenging ROS more efficiently than Mn(II) (Tang *et al.*, 2013) or by competing with Mn(II) for binding to the enzymes responsible for Mn(II) oxidation (Tojo *et al.*, 2020). Previous studies also employed EDX ana­lyses to characterize bacterial or fungal BMOs and the adsorption of other heavy metals on them (Zhou *et al.*, 2015; Kim *et al.*, 2016; Yokoo *et al.*, 2022); however, only some performed detailed distribution ana­lyses of each element. A physicochemical study on fungal BMOs by Luo *et al.* (2019) reported the adsorption of Co on BMOs produced by *Pleosporales* sp. using TEM-EDX elemental mapping. The present study provided clear evidence to show that dissolved heavy metals, such as copper, were adsorbed and immobilized on BMO precipitates rather than directly associating with hyphal surfaces using elemental distributions in fungal hyphae-BMO assemblages. Based on the known characteristics of poorly crystalline or amorphous BMOs that adsorb a number of metals as well as previous findings, the adsorption of metals to the BMO nodules of strain TS-2 may occur in other heavy metal species, such as Co, Ni, or Zn. Groups of fungi are known to tolerate heavy metal stress by producing extracellular chelating substances to chelate metals, thereby reducing their uptake (Cervantes and Gutierrez-Corona, 1994; Baldrian, 2003; Bellion *et al.*, 2006). Therefore, although remaining inferential, heavy metal adsorption to BMO nodules may enable strain TS-2 to control the concentrations of metals in the local fungal microenvironment, thereby aiding in the acquisition of trace elements and/or prevention of excess metal uptake.

## Conclusion

A new *Periconia* isolate, strain TS-2 was confirmed to oxidize and immobilize dissolved Mn(II) as BMO precipitates, which has not yet been exami­ned in detail for *Periconia*. Notably, the BMOs produced by strain TS-2 formed characteristic distinct nodules on hyphae, which further sequestered dissolved Cu(II), as visually evidenced by SEM-EDX elemental mapping. The formation of these BMO nodules appeared to play a role in preserving larger areas of fungal hyphae, preventing encrustation and potentially maintaining hyphal functionality. The present study additionally served as the first genomic characterization of‍ ‍a‍ ‍Mn(II)-oxidizing member of the genus *Periconia*, identifying the presence of multiple genes encoding laccases that are potentially involved in Mn(II) oxidation by this strain. Further investigations of this isolate and other *Periconia* strains will reveal the mechanisms underlying their biomineralization behaviors and increase our understanding of their ecological roles and significance in the natural geochemical cycles of heavy metals.

## Citation

Tsushima, S., Nishi, Y., Suzuki, R., Tachibana, M., Kanaly, R. A., and Mori, J. F. (2024) Formation of Biogenic Manganese Oxide Nodules on Hyphae of a New Fungal Isolate of *Periconia* That Immobilizes Aqueous Copper. *Microbes Environ ***39**: ME23102.

https://doi.org/10.1264/jsme2.ME23102

## Supplementary Material

Supplementary Material

## Figures and Tables

**Table 1.** Relative compositions (by weight% and atomic%) of abundant elements identified in the observed field of the SEM-EDX ana­lysis shown in Fig. 5.

**Element**	**wt%**	**at.%**
C	77.04	83.36
O	17.05	13.85
N	2.04	1.89
Mn	3.18	0.75
Cu	0.69	0.14
**Total**	**100.00**	**100.00**
