# Supplementary material for: Formation of Biogenic Manganese Oxide Nodules on Hyphae of a New Fungal Isolate of Periconia That Immobilizes Aqueous Copper
Source: Microbes Environ. 2024 Jun 12;39(2):ME23102. doi: 10.1264/jsme2.ME23102 (PMC11220447; doi:10.1264/jsme2.ME23102)
Supplement: Supplementary file 1 — Supplementary Material [file 39_23102_s1.pdf]

Supplementary information for

Formation of biogenic manganese oxide nodules on hyphae of a new fungal isolate of *Periconia* that immobilizes aqueous copper

Table S1. Predicted laccases / multicopper oxidases encoded in the genome of *Periconia* sp. strain TS-2.

Scaffold	Predicted CDS	Sequence	Protein BLAST results
NODE_1<br>(2,357,370 bp)<br>(JASNQO010000001)	1109576..1109662,<br>1109715..1109767,<br>1109814..1110039,<br>1110154..1110260,<br>1110348..1110902,<br>1110963..1111179,<br>1111227..1111317,<br>1111375..1111685	MLSLLLLWACFHVSWAKTVYYDFNIGWVSRAPDGFSRPVIGINGQW<br>PIPTIEADVNDTIVVTAHNSLGNETTSLHFHGMYQRETSISDGPVGVT<br>QCPISPGQSFTYTFTAWPAGTHWYHSHNKGQYPDGLRGKMIIHNPE<br>WEKSLNIDEQIPLSISDWYHTEMPYLIHDFLNVKNAGGHLPVPNSFL<br>VNDTSNPPCINFEPKKRYLLRIVSHSAIACGLFYITDHNLTVVAVDGQ<br>EVHPRDTDKIIICAGQSYDVVVTGHSNPKSGIQYIAKMTPDMLTLPP<br>PPEDKITLRGSIKYTKGGQQLEASQQPSTSNFLDDVSLQPLDNEPLL<br>QNPSKWIYFRTNQRFYENIGTRIGLGQEPWVEPKVPTLYTALSTNDN<br>AFTASTYGPGVAPEIVKYNEVVQIHMQNLQPFAHPMHLHGHVFQVA<br>ARGKGSWDGKESSLHPIPMKRDTVVIPPEGYLVLRFHAWNAGVWL<br>FHCHIDFHLVGGMAASIIEAPDVLQRQQTIPESGSLLCTANRQGAFG<br>NCAGQSGQISAADAAKQCNTVLNSNGESYGALVT (548 aa)	Multicopper oxidase<br>[*Trematosphaeria petusa*]<br>(XP_033685137) 63.59%<br><br>Multicopper oxidase<br>[*Lentithecium fluviatile* CBS 122367] (KAF2680798) 63.21%<br><br>Ferroxidase [*Periconia macrospinosa*] (PVH95171) 61.43%
	1459898..1461097,<br>1461149..1461826	MLSFACRAFTFVTCLLGYSSIRNFNLKSYFGNGQFSSTVAVQFEIHLT<br>HGQVNPTGAGFREAILVNGTFTGPTLRLNTGDQVEFLVRNYLRKDT<br>TIHFHGISQAASPWVDGVPGVSQHPIRPGSSYLYKWKAEDSGVYFY<br>HAHSRGQMMDGLYGAIVVAPKMRVDRPFHMISRDPSDYAAMREAE<br>GKLQTLMISDWSQFSFDEFYAIEREANIDFTCMDSIIVNGAGSQYCL<br>DRPSLDDYTDPLVKAILASTGQKGITDKGCVPPIQLFQGNFSLHLDT<br>LPAKSYRECVPGVGGHANFTVPVHSGEKWAALTFINTGGLYPLKVTI<br>DNHPLHVYAVDGQYIYPQKVDQLIVTNGERYSVFIKLDQQVGKYTI<br>RTANNLLGQVLGGFAALSYNGATDDPPDPKPLMNYAGHSIIPDVRLF<br>SDLDARPYPPIHPAKAVDRTHKFLIKKLGRPYGAYEWTLSGGEGYN<br>MSEENRESPLLFQSPDRINFSELVLRNRKGEWVDLIIEIEGPFAQAHP<br>MHKHGSKAFLIGQGVGAFPWVSVQDATKELPLGTFNTVDPPFKDTF<br>KTLEAVNNNAWLVLRYKAEAAGAWLFHCHIQTHLTGGMGVVLLD<br>GVNRFPQVPEEYKEWNGFKQPR (625 aa)	Multicopper oxidase<br>[*Melanomma pulvis-pyrius* CBS 109.77] (KAF2795494) 81.22%<br><br>Multicopper oxidase-domain-containing protein [*Paraphoma chrysanthemicola*]<br>(KAH7094402) 70.90%<br><br>Multicoppe oxidase<br>[*Viridothelium virens*]<br>(KAF2230543) 68.96%
NODE_4<br>(1,487,738 bp)	877158..877299,<br>877355..878021,	MVAFRFAMAVTWLMAMPATVYPILIPKPVGKRAACAGNTGDDRAS<br>WCDFSIDTDWYEEVPDTGVTREYWLELLETEISPDGFTRSAMTVNG	Laccase-like multicopper oxidase<br>[*Periconia macrospinosa*]

(JASNQO010000004)	878072..878975	TVPGPTITADWGDELVIHVVNNLNVNGTSIHWHGLRQNYTNQNDG<br>VVSITQCAQAPGDTYTYRFRATQYGTTWYHSHFSLQAWDGIFGSIII<br>NGPATANYDVDVGPIIISDWFHESVYSLASQAETAGPPTPENGLINGT<br>NVFGDDNSTDQTGSRFELEFQSGSSYRLRLINVAIDSFWKFTIDNHTL<br>TVIAIDLVPIEPFTTDIVSIGIAQRYDVIVNADQADVASDFWLRFIPQS<br>ACSSNDMADNIRGIIHYDGSTGTPSTAQYEFTDECVDMDSSLLVPHV<br>QKDVPADHFYQEQEDVGLAFTTEGTLRWTINGSTLAVQWGDPTVL<br>QIMNNDTNFDTSQNLIRLDEAEQWAYIIIETSIGVAHPIHVHGHDFYL<br>LAQGTGTFSSDIALTLNNPPRRDVAMLPANGFLVLAWETDNPGAWL<br>VHCHIGWHTVQGLALQFAERIGDIPSVIDSDSLGETCNNWDTFATQN<br>NIVTDDSGV (570 aa)	(PVH92490) 79.61%<br><br>Multicopper oxidase [*Lentithecium fluviatile* CBS 122367] (KAF2691773) 79.41%<br><br>Multicopper oxidase [*Clohesyomyces aquaticus*] (ORY13561) 76.83%
NODE_7<br>(1,424,036 bp)<br>(JASNQO010000007)	1201594..1202010,<br>1202067..1203103,<br>1203161..1203596	MRLNRIVGVCLLAYATLSAAEIPENHVLWGEEGPPLSLQKRATSSSSP<br>SKSSSTLSKTSSSSSTSSPSASSSKAADPNCSNGPFTRTCWSNGYSVA<br>TDFDTKWPTTGNTAHYNLELTSGTCNPDGHGDRPCQLFNHQYPGP<br>VITANWGDTISVTLKNSLQANGTGIHWHGLRQLKTNPQDGVPGVTE<br>CPLAPGDTKTYTFKATQFGTTWYHSHFSAQYGEGAFGAMVINGPAS<br>SNYDVDLGPYLINDYYYETAWQIGVTSHNNLQLGKAPPTPDTFVIN<br>GTNKNSGGGGSYNQVKNLQKGKKYRLRLINASLDNSIKVSLDNHN<br>FTVITNDLVPVKPYNASWVLLGIGQRYDVIFTANQAADNYWFRVEA<br>ATDCASANNYYGRSIFSYAGASGGTPNSQGSSAPAGCTDPKSVPWV<br>KNSVDRSAFVSQARELDVDLTVAQTTTNQQNIVVWSVNLTGIDVEW<br>DKPTSLYVKDHNTSYPSTYNLIELPKEGIWTYWIIQEPKGTKVPIPHPI<br>HLHGHDFYILGQGTGVFDKDSSPDTMTYDNPPRRDVAFLPGGGWL<br>ALAFPTDNPGAWLMHCHIAWHVADGLAVQFLEGKDSMTLGDGAW<br>DKTCSNWAKYQKTMPYSKMDSGLKMMMKE (629 aa)	Putative multicopper oxidase, type 1 [*Lindgomyces ingoldianus*] (XP_033548708) 65.98%<br><br>Putative multicopper oxidase, type 1 [*Clohesyomyces aquaticus*] (ORY12815) 66.13%<br><br>Putative multicopper oxidase, type 1 [*Massariosphaeria phaeospora*] (KAF2874552) 66.11%
	1282240..1282324,<br>1282422..1282957,<br>1283011..1284084	MLSRNLIFASAALVGFAEAFPSLQPTHLGKRATCSGNTASTRSEWCD<br>YSIDTDYTSEAPDTGVTREYWLELTDVTVAPDGVSRSAMAVNGTIP<br>GPTLFADWGDTVVVHVTNSLTDSQNGTSIHFHGIRQNYTNDQDGVT<br>SITQCPTAPGSSLTYTWKAVQYGSTWYHSHFALQAWEGIFGGIVING<br>PATSDYDEDLGMLFLNDWDHQTVDELYISAETDGPPTLDNGLINGT<br>NTYDDGGYRFNTSFTSGTSYRLRLVNAAVDTHFKFMLDNHTMTVIA<br>SDLVPIEPYNATVLDIGMGQRYDVIITADQADVADNFWMRAIPQSAC<br>SENDNEDDIRGIVYYGDSAGTPTTSAYDYDDSCDDETDNITPYISKT<br>VGSDVSQTDEEAATVAYNDDNLFRWYLNSTTMEVSWTDPTLMQIL<br>NGSTTYETDDAVITLPDADVWVYLVITTTLAVPHPIHLHGHDFYVLA	Multicopper oxidase [*Trematosphaeria pertusa*] (XP_033683068) 85.66%<br><br>Multicopper oxidase [*Lindgomyces ingoldianus*] (XP_033546087) 80.04%<br><br>Multicopper oxidase [*Lentithecium fluviatile* CBS

		QDTGTYSSSVSLNLDNPPRRDTAMLPASGYLVVAFETDNPGAWLMHCHIGWHTSEGFALQFVERYDEIAALTDSDTLESNCDAWETYQDDNSIEQEDSGV (564 aa)	122367] (KAF2678339) 79.61%
NODE_8<br>(1,404,679 bp)<br>(JASNQO010000008)	1300597..1300748,<br>1300810..1301106,<br>1301165..1302079,<br>1302128..1302341,<br>1302397..1302410,<br>1302458..1302698	MGLLKYSVISLLTLLGTTPLASARSLRNTIIPRQASSGFQCTYPAGWESCNDKNNRRCWVKDPSGKTYDIDTPYETDTPTGIERVVNLEVEEADISPDGFKRTAQLINGTYPGPLIEACWGDTLVVNVKNKIKHNGTTIHWHGLRMLNENQMDGVNGVTQCPIADGDTFTYRFHLKQYGHTWYHSHYSAQYTDGVAAPLLIHGPNTAEWSEEWSPIIVADWYHQNAYEAMHQALTGPPTAAAIVVNGTGRNNKDNTGKYFEQTFKRDESYLIRVINGGTDFHFHFSIDDHELQVVSADLVPIKPFTTKTLAVGIGQRYGVIVKANATAAKNGKYWMRTEYDGSSCNPNHLNFPYTDLDKQRVGIISYEDAGQGDPTSERWGGYVGCADPVIDPHIKWSVPNPTAAQMAPHYAGLDLVQSHKTHGFSRWELAETPLWINFSDPSIVHTDDKTWNPPEYALESYNAKEAGGFVYMLINSGNGSRTNMFAGSHPIHLHGHDFAVIKNGTGIFDPTNPGWNMPSNPPRRDVAMLPNNGHIVLAFKTDNPGIWLVHCHIGWHAGSGLALQVVERQSEISKTIGSIENVTNNCKKWTDSVNGPKNWDYSWQDDSGI (610 aa)	Multicopper oxidase [*Lentithecium fluviatile* CBS 122367] (KAF2675871) 58.41%<br><br>Multicopper oxidase [*Trematosphaeria pertusa*] (XP_033682372) 63.08%<br><br>Laccase-like multicopper oxidase [*Periconia macrospinosa*] (PVH98835) 57.21%
NODE_9<br>(1,290,941 bp)<br>(JASNQO010000009)	51710..51984,<br>52044..53420,<br>53477..53639	MKWISSLLNLAWFSSGALAYNVQTHDASFTPDIVLRVALENTELHCRTRLTTMVNGTVPGPELRIPEGRTTWIRVYNDMADENTTIHWHGLTQSVAPFADGSPLASQWPIAPFHYFDYEIHPEPGSAGTYFYHSHVGFQAVSAAGALIVEDPEAPPYEYDEEKVIFLSDFWNKTDQNITTGLAQNPLGWIGDVDGLLVNGYGRPNTSTSTLESCTISPISIDPGKTYRLRFIGSSGMTFFSLAIEGHDEMTIIEADAVYSKPINTSYLQIATGQRYSVLLRTKTEEELKAEGKELYYMQLKTLERPIIYTGFAAFTYSKQPSLPIKPPTVSKWPLNLPSTTYGFLDYELTPYKVTQKVPSRAEVDRTIVLKVYQTNPLSDLNPFNQTFWTVNNYTPWFEHTAKKPYLLSMYEDDSSYLPNYKTALQQGGFDPKAKAFPLKIGEVVDIILQNVGMGPLTTQGSNSMDMHPFHFHLSHYWDMGSGNGTYDPVAHDARLEEAYALGWSPPLRDTSQLFRQPNDHQALGVDTNWRAWRLNVTTPGAWMMHCHTLVHMVMGMQSVWVAGDKDDLTPIPQQLASEFYTYGGGAFGNTTHAPRMVHFFDEK (604 aa)	Multicopper oxidase [*Piloderma croceum* F 1598] (KIM76294) 47.69%<br><br>Multicopper oxidase [*Stipitochalara longipes* BDJ] (KAE9377494) 48.49%<br><br>Laccase, putative [*Paecilomyces variotii* No. 5] (GAD97655) 47.51%
NODE_14<br>(983,845 bp)<br>(JASNQO010000014)	55504..55623,<br>55680..55904,<br>55951..56970,<br>57026..57145,	MVFKYAFLIFSLLQSVVVGEVREYNLTLEPKWLSLDGNSRPVLTINGQTPGPVIEGEEGDTLRITVTNKMFIEATMHYHGVFQVDKYWNDGVPGVTQWPIEPRNSYTYEFTLTNQTGIYFYHGHFGPAFADGQRGPLWIRPKASRPRPYSLISSKDSDIKEMLDAESQPHHLLVSDWNYDGMEILTV	Putative multicopper oxidase, type 1 [*Corynespora cassiicola Philippines*] (PSN69530) 84.91%

	57195..57234, 57287..57413, 57469..57574	MFRDAGVTPNCAASIVTNGKGRITCLDPEVIKKYNKHGPRDDMACLIPRHGAEFMNKRTCKESNTDFEVIQAKDGQKYLFINFIHPGAHHELRISVDEHDMWLVAADGDFTKPKKVQAFNLNMGDRISVLVPLDQKPRDYAIRLASLAPEQIIQGLSILRYPNQPDARDKHGVMLTPSSKPHIDLVGNMISNGIAMDELTDLSPFPDRQPPATSDTTLRFVANQTSPSTWVLASEPHQGFRQQMPPILWNEESRGPTTFSGLKNGSVVDIIYENGAYAMHPFHKHNHKAFIIGRGDGYFRWPDVATALKKTPENFNMVDPPLRDGARLAAGPGSWTVIRYTITFPAMSMLHCHRIAHFAGGQQIVLVEGGDTMPPAPDYIRHLTHSEFVPPLRYGPLD (585 aa)	Multicopper oxidase [*Viridothelium virens*] (KAF2236996) 70.77%<br><br>Multicopper oxidase type 1 [*Botryosphaeria dothidea*] (KAF4306496) 74.38%
	614158..614328, 614394..615043, 615097..615160, 615217..615512, 615564..615651, 615706..615837, 615895..615999, 616050..616277	MAFLRTLFCLVAAVAAVPAPQAETSSASASASASVTSSAAGSACTGNTASDRTTWCDYSIDTNYYDEVPDTGVTVEYWFEVTNITAAPDGVERVVLAVNGSVPGPTIEANWGDTLKIHVTNSLTNNGTGIHWHGIRQNWTNQEDGVPSITQCPIAPGESYTYTWRATQYGTSWYHSHYSLQAWNGVFGPMVIHGPATANYDEEFGTVMLSDWTHQTADEKYTYAQTVGPPLLDNGLINGVNTYEGGGSRFEGSFEAGKSYRIRLINTAIDTHFKFAIDNHTFTVIANDFVPIVPFEADYLDITMGQRYDIIVSADQPVSDVTDYWMRAVPQAGCSSNNNSADIRGIIRYDSSSTADPTTKAYDALSDDTCVDVDLSNLVPHVSQTVVNPSDDSDLTVSVAKNSAGLFKWQIGLNSMLVQWDNPSLLQIYDGNATFESEENVYTLPDANTWVYFVISSAIPVPHPIHLHGHDFFILAAQDNADFDSSVTLNFDNPPRRDVANLPTAGYLVIAFLTDNPGAWLMHCHIGWHTSEGLALQFVEREDEISALLNTDTLQDTCSAWNSWADSTGIEVEDSGV (577 aa)	Multicopper oxidase [*Lentithecium fluviatile* CBS 122367] (KAF2687906) 80.11%<br><br>Multicopper oxidase [*Trematosphaeria pertusa*] (XP_033675857) 74.44%<br><br>Multicopper oxidase [*Karstenula rhodostoma* CBS 690.94] (KAF2445480) 72.15%
NODE_26 (611,559 bp) (JASNQO010000026)	543826..544047, 544181..544393, 544556..544633, 544696..544754, 544804..545088, 545222..545298, 545347..545492, 545601..545791, 545841..545937, 545990..546064, 546115..546486	MRSALNLGASLLGLCCTLVNGSPLRAEPLGFSPALSLEKRQDCTNGPTSRNCWLPGFDADTDMYSSWPDTGRTVTYDLTITNTTCNPDGAQSRVCMLINGKMPGPTIIGNWGDTFKITINNNLQHNGTSIHWHGLRQLNSNSQDGVNGVTECALAPGDSKTYTFRATEYGTTWYHSHFSAQYGDGVIGTIIINGPATANYDLDLGTYTVQDWYHITAFQAARRAFESGQLFGGPPTGDNILINGTNKSPDGSTGSYNNVQLTPGKKHRLRIINPSLDIAMRVSLDGHPFQVIANDLVPVVPYDTNYILIGIGQRYDVIINANQSSGNYWFRAEAENACVSFNNGVGRSIFTYENSTVADPTTDALPGNPADCNDPTPTPKIVKNVPSASFASQAQELPVAFGNTTVASNNQSLILWTVNGTSMVIDPAEPTLEYLAKDNTSFPPNYNLVSVSPNAAWTFWVIQQAANAPPLAHPIHLHGHDMYVLGKGSGQFDVDQHLSSLTFNNPPRRDVTHLPAGGWLVISYPTDNPGAWLMHCHIAFHVAMGLSNQFLERPSEINIPPSGDEWWDTCANYEAYVSGNPIYPQDDSGLKKRWPPSTYAFDS	Multicopper oxidase [*Lentithecium fluviatile* CBS 122367] (KAF2687655) 79.80%<br><br>Multicopper oxidase [*Trematosphaeria pertusa*] (XP_033675639) 73.49%<br><br>Laccase precursor [*Bimuria novae-zelandiae* CBS 107.79] (KAF1979684) 73.52%

		M (604 aa)	
NODE_27<br>(604,823 bp)<br>(JASNQO010000027)	534309..535353,<br>535410..535510,<br>535559..535610,<br>535658..535840,<br>535892..536139,<br>536196..536248,<br>536304..536391	MRVQSHILACAWSVASCVSAAGLTQKQTNGNSLLGTLLAPLLNGDA<br>AAWIGKTIENTNVYTSSPNTGKEVKYTFDVTRGFLAPDGVNASMLL<br>VNGAFPGPTLEANWGDTFVITVNNKIQGPEAGTSIHWHGILQKESQ<br>WMDGVPSVTQCPIIPGGSFTYRFKADRPGTSWWHSHYAAQTAGGL<br>HGAMIIHGPCQKSTEYDIDIGPVVLEDHYHIDYHELIGGYTSVPLQLI<br>YSDSNLINGKGEYDCSKTDRQCTPNAGYSKFKFQSGKRHLLRLINA<br>GAEALQHFTIDNHEFQVIELDYVPITPYTTKKITLSPGQRANIVVKAT<br>GKPTDTVWMRSDIDPNCAAASNPKAQAIIYYEKANTNGTPNTTATP<br>YTVGGCATEPIANTVPVYPLTPPANPATDVDLDITVGFNSTGHMLWY<br>MNNSTFFADTSRGLLFDAQESAHPNFSNPLWNVWNFGSNSSVRML<br>MRNYHITPHPMHLHGHDFWVLAEGHGEWDGTVVNKNNPIRRDTH<br>QLLPMDGDKPGYAVIEWTQDNPGMWAFHCHIFSHSSLGFYINVLEQ<br>PDKIRSLNIPSANKDTCKIWNDWAKTHGVDQPDSGV (589 aa)	Multicopper oxidase [*Melanomma pulvis-pyrius* CBS 109.77] (KAF2792949) 69.72%<br><br>Multicopper oxidase [*Zopfia rhizophila* CBS 207.26] (KAF2183914) 56.65%<br><br>Cupredoxin [*Exophiala* sp. JF 03-3F] (KAI1620260) 53.45%
NODE_28<br>(580,671 bp)<br>(JASNQO010000028)	187120..187371,<br>187422..187508,<br>187563..187897,<br>187949..188214,<br>188307..188731,<br>188783..189196	MLYTFTKVIVLIGLLCAPVLSSPADFSYLKPDVPWKNKKRGLLERDG<br>YGTGCENGPHSRSCWSGGLDIDTDMDEHWPTTGKTVKYDFEITNM<br>TLAPDGLSRPMAVINGQYPGPAIVADWGDTIEVTVTNSLTTNGTGIH<br>WHGLRQMGSNEMDGVGGLTECPIPPGGKTVYTFKATQYGTSWYHS<br>HYSVQYGDGLVGPIVIHGPATKNYDIDLGALPFTDWFHKPLFEVNA<br>AALHANGPPVADNLLVNGSMTSSFGGKYSVTTLKKGKSHRLRLVNT<br>GINNWVHVALDGHPFTVIAADFVPVVPYKADSLSIAVGQRYDVIFH<br>ANQTVGNYWLRVGTGGGQCDGPNSNSANIGAIFRYAGAPNENPTSS<br>ASTTLPTGCYDESGVIPYVKTQVPRETPDQMTLGFSNTGATGNLIQ<br>WLIDGTPIRVDLEKPTLQQISDGNDTYNKNENVFLVGEKDKFQYWV<br>IQQNQSIPAALPHPIHLHGHDFYVLDQQAGATWSGDTSRLKMDNPIR<br>RDTATLPAKGYLVLAFESDNPGVWLMHCHIPFHVSAGFGVQFVERR<br>DEIIGSIGSLDNFQAQCKDWSKFQADKYPDGFTEGDSLL (592 aa)	Multicopper oxidase [*Trematosphaeria pertusa*] (XP_033682011) 71.55%<br><br>Multicopper oxidase [*Melanomma pulvis-pyrius* CBS 109.77] (KAF2794437) 68.80%<br><br>Multicopper oxidase-domain-containing protein [*Massariosphaeria phaeospora*] (KAF2873996) 67.54%
NODE_33<br>(429,961 bp)<br>(JASNQO010000033)	381567..382396,<br>382460..382604,<br>382657..382857,<br>382906..383030,<br>383085..383128,<br>383302..383353,<br>383404..383433,<br>383480..383516,	MLLNTVACWLSLSSLALGGSIPRKADGLPPSRVVVERQATTSTQVPD<br>GQCTNGPLTRNCWSSGFSISTDFDAKAPPAGKTVTYNLEITNTTLSP<br>DGGAERPVMLINNQYPGPVLRASWGDTLIVNVKNSLQDNGTGIHW<br>HGVRQLNSCQHDGVPGVTECPIAPGKTRQYVFKCTQFGTTWYHSH<br>WSAQYSEGTLGTLIIDGPATANYDEDLGTLPLAEWYYAPGFTLNEQ<br>AQHSQVGPPKPDNILVNGTHKNKDGGGSYFKMKVKKGKKYRIRIIN<br>TSVDTTFSVSMDKHPFTVITSDFVPVKSFRAEQLTLAIGQRYDVVIN<br>ANQTVDNYWFRVSVGTDCGSNSILDAGIPLGAILQYDGASNAEPTS	Multicopper oxidase [*Trematosphaeria pertusa*] (XP_033690608) 86.78%<br><br>Multicopper oxidase [*Lentithecium fluviatile* CBS 122367] (KAF2691053) 83.25%

	383566..383628,<br>383677..383922	TGVTMRTTCTDETNLVPFVPNSVPTSLVPEAEMKLNHHQDANDNFL<br>FRWTIDGTPIIVDWNDPSLETALSGSSDFGNNSNVFEMDSNKWYFW<br>WIQTTTPIALPHPIHLHGHDFYILGSGPGTWDGSTSGLNFQNPTRRD<br>TATLPAGGYLLLAFPADNPGTWVMHCHIAWHASQGLSVQFLERKSE<br>IKGAIGSTTGFEQGCKEWDNYWFPGNHPYNQTDSGI (590 aa)	Cupredoxin [*Massariosphaeria phaeospora*] (KAF2869685) 78.17%
NODE_35<br>(419,397 bp)<br>(JASNQO010000035)	387125..387823,<br>387879..387948,<br>388006..388796,<br>388844..388888,<br>388939..389322	MKLVERFWTAVSCLLNILTPSPFSHELGDGIQQLPLFDQGVHPAAKP<br>GPIFRPPGRLRRGSNDNFQCDYTAMTGWTNCSTPSTRGCWLENKST<br>GEMRDVNTNYELITLTPMGITRNYTLNVTKAGNINVDGLDFIGGFM<br>FNDKYPGPWIQACWGDTLSITVNVDPEFEMGTSVHWHGIRQWLTM<br>HMDGVPGITQCPIAPRSSFTYEFKAMQYGSSWYHSHYSLQYADGLL<br>GPLTIHGPSSDNWDIAPAKPLFISDWNHNVVTGTDDVANNRTVLLG<br>GIGNMTRSYPDAVVNITEHHDPFNLTFVEGTKYLLRIINVAYDSTFLF<br>SIDNHNFTVISADFVPIEPYTTTSIAVGIGQRYNIIVDANPLESNATDF<br>WIRTHILNGTNCHTGGPPSGQYYMQTGIIRYDESSQADPTTTQWPNL<br>DTTYCRDEPAFTPIVKWEPKAPANPSEPIRIIQFGAATDKTVINPGSFS<br>FLTPAELNANPQIRVPLRIDWQNITFLNLNNKDGWNDSFVILPEQYK<br>ATEWIYLVITNSEENRGHPIHLHGHDFAIMDVAANKAFNASEPIIIKD<br>NPPRRDVVYLPENGYVVIAFKADNPGAWLIHCHIAKHASMGLAMQ<br>VLEDRDGADARWPAHISPAWNRANDLCTAWKDWCSETKWGNCTN<br>LFIQDDSGI (662 aa)	Laccase-like multicopper oxidase [*Glonium stellatum*] (OCL07909) 49.05%<br><br>Cupredoxin [*Leptodontidium* sp. MPI-SDFR-AT-0119] (KAH6699784) 47.57%<br><br>Laccase [*Lachnellula suecica*] (TVY71373) 46.66%
